# Perception of health professionals on extramural research funding in India

**DOI:** 10.6026/973206300213760

**Published:** 2025-10-31

**Authors:** Mayank Das, Sanchit Pradhan, Supurna Pandit, Arunendra Singh Chauhan, Baidnath Jha

**Affiliations:** 1 Department of Public Health Dentistry, Subharti Dental College and Hospital, Swami Vivekanand Subharti University, Meerut, Uttar Pradesh, India; 2 Department of Public Health Dentistry, Maharishi Markandeshwar College of Dental Sciences and Research, Mullana-Ambala, Haryana, India; 3 Department of Microbiology, Sarjug Dental College and Hospital, Darbhanga, Bihar, India

**Keywords:** Extramural research grants, funding agencies, health professionals, research proposals, India

## Abstract

Limited awareness of extramural research funding hinders healthcare research growth in India, especially among non-medical professionals. This cross-sectional study among 400 health researchers assessed awareness and challenges related to research grants. Only 48.6% knew of funding agencies, with 82.9% recognizing only ICMR and 74.3% unaware of proposal drafting formats (p=0.001). Most participants (85.7%) lacked experience with extramural grants. Thus, we show the urgent need for research grant training and integration of proposal writing into health curricula to enhance funding utilization in India.

## Background:

Health research funding has been steadily increasing throughout the globe, with an estimated total investment of over $265 billion annually in health research and development. However, India's presence in this global forum remains disproportionately small despite having the second-largest population in the world and a significant burden of disease [1, 2]. Research funding is crucial for advancing medical knowledge, improving healthcare delivery and addressing public health challenges. Extramural research grants, in particular, provide essential financial support for researchers to conduct independent studies outside their institutional budgets. The landscape of health research funding in India is complex, with multiple governmental and non-governmental agencies offering financial support for research projects [3]. The Indian Council of Medical Research (ICMR), Department of Science and Technology (DST) and Department of Biotechnology (DBT) are among the major government funding bodies, while several international organizations also provide research grants [3]. Despite the availability of these funding opportunities, there appears to be a significant gap in awareness and utilization among different health professional communities in India. Previous studies have indicated that while the medical community is relatively aware of funding agencies, other health professionals, particularly those in dentistry and allied health sciences, are missing out on millions in funding opportunities [3, 4]. This lack of awareness and participation not only limits individual research careers but also hinders the overall development of health research in the country. The situation is particularly concerning given that India faces a dual burden of communicable and non-communicable diseases, with oral health conditions being among the most prevalent yet neglected health problems [5, 6]. Research has shown that awareness and knowledge about research funding opportunities vary significantly among different health professional groups. A study conducted among postgraduate medical students in New Delhi found that while 78% of participants considered research important, only 42% were aware of funding opportunities [7]. Another nationwide survey on barriers for dental research in India identified lack of funding and lack of training in research methodology as major obstacles [4]. These findings suggest a need for comprehensive assessment of awareness and perceptions regarding extramural research funding across various health professional disciplines. The importance of extramural research grants extends beyond individual research projects. These grants play a crucial role in building research capacity, fostering innovation and addressing national health priorities [8]. They also provide career development opportunities for researchers and contribute to the overall advancement of health sciences. In the context of India, where health research funding remains limited compared to the country's needs, maximizing the utilization of available extramural funding opportunities is essential [3, 2]. Despite the significance of this issue, there is a paucity of comprehensive studies examining the perception of health professionals regarding extramural research funding opportunities in India. Most existing studies focus on specific disciplines or regions, limiting the generalizability of their findings. Furthermore, there is limited information on the challenges faced by health researchers in drafting grant proposals, which is a critical step in securing research funding. Therefore, it is of interest to assess the perception of healthcare researchers regarding funding opportunities of extramural research grants for health researchers in India and to identify the challenges faced by health researchers in drafting grant proposals.

## Materials and Methods:

## Study design and setting:

An observational cross-sectional study was conducted by the Department of Public Health Dentistry, Subharti Dental College and Hospital over a period of 3 months. The study aimed to assess the perception of healthcare researchers regarding funding opportunities of extramural research grants in India and identify challenges faced in drafting grant proposals.

## Sample size and sampling technique:

The total sample size for the study was 400 participants. The sample included health professionals from various disciplines, including medicine, dentistry, pharmacy, nursing and allied health sciences. Participants were selected using a convenience sampling technique from different healthcare institutions across India.

## Inclusion and exclusion criteria:

Health professionals including faculty members, postgraduate students and researchers working in healthcare institutions were included in the study. Participants who were unwilling to provide informed consent or those who did not complete the questionnaire were excluded from the final analysis.

## Study tool:

A self-administered structured questionnaire was developed for the study, containing 15 closed-ended questions. The questionnaire was designed in both English and Hindi languages to ensure better comprehension and wider reach. The questionnaire was divided into three main sections:

[1] Demographic details: Age, gender, professional qualification, field of specialization, years of experience and institutional affiliation.

[2] Knowledge and awareness: Questions about awareness of funding agencies, knowledge about extramural research grants and understanding of grant proposal drafting.

[3] Practices: Questions about previous experience with grant applications, frequency of searching for research grants, time taken to draft proposals and challenges faced in the process.

The questionnaire was validated by a panel of experts including senior researchers and academicians from various health disciplines. A pilot study was conducted on 30 participants to assess the clarity and feasibility of the questionnaire. Based on the feedback, minor modifications were made to improve the comprehensibility of certain questions.

## Study procedure:

The questionnaire was distributed to potential participants through email and WhatsApp. A cover letter explaining the purpose of the study, assurance of confidentiality and voluntary participation was sent along with the questionnaire. Weekly reminders were sent to the participants to encourage completion and submission of the questionnaire. The data collection period spanned 3 months to ensure adequate response rate.

## Ethical considerations:

Ethical clearance was obtained from the University Ethical Committee of Subharti Dental College and Hospital (SMC/UECM/2023/629/296). Informed consent was obtained from each participant before their inclusion in the study. Participants were assured that their responses would be kept confidential and used only for research purposes. They were also informed that their participation was voluntary and they could withdraw from the study at any time without any consequences.

## Statistical analysis:

The collected data were compiled and entered into a spreadsheet computer program. The data were then exported to the data editor page of SPSS version 20.0 for statistical analysis. Descriptive statistics including frequencies, percentages, means and standard deviations were calculated to summarize the data. The chi-square test was used to compare categorical data between different groups. The level of statistical significance was set at p<0.05.

## Results:

A total of 400 health professionals participated in the study. The mean age of the participants was 35.6 ± 8.2 years, with 58.3% being female and 41.7% male. Regarding professional qualifications, 42.5% were postgraduates, 35.0% were undergraduates and 22.5% held doctoral degrees. The participants represented various health disciplines, with 32.5% from medicine, 28.0% from dentistry, 15.0% from pharmacy, 12.5% from nursing and 12.0% from allied health sciences. The mean years of professional experience were 10.3 ± 6.7 years. When asked about their knowledge of extramural research funding agencies in India, only 48.6% (n=194) of participants responded positively, while 51.4% (n=206) were unaware of such agencies. Among those who were aware, 82.9% (n=161) could name only the Indian Council of Medical Research (ICMR) as a funding agency, while the remaining 17.1% (n=33) were aware of other agencies such as the Department of Science and Technology (DST), Department of Biotechnology (DBT) and Council of Scientific and Industrial Research (CSIR). The differences in awareness levels among different professional groups were statistically significant (p=0.001). Medical professionals showed the highest awareness (68.3%), followed by dental professionals (42.9%), pharmacy professionals (35.0%), nursing professionals (30.0%) and allied health professionals (25.0%).

Regarding opportunities to work on extramural grants, 85.7% (n=343) of participants reported never having had such opportunities in their research institutes, while only 14.3% (n=57) had experienced working on extramural grants. The differences between professional groups were statistically significant (p=0.001), with medical professionals reporting more opportunities (25.0%) compared to dental (12.5%), pharmacy (8.3%), nursing (6.7%) and allied health professionals (4.2%). When asked about their knowledge of the pattern of drafting extramural research grants, 74.3% (n=297) of participants reported not knowing the proper pattern, while only 25.7% (n=103) were familiar with the grant proposal drafting process. The differences among professional groups were statistically significant (p=0.001), with medical professionals showing better knowledge (40.0%) compared to dental (21.4%), pharmacy (16.7%), nursing (13.3%) and allied health professionals (12.5%). Regarding awareness of career opportunities related to funding agencies, 62.9% (n=251) of participants were unaware, while 37.1% (n=149) had some knowledge. The differences among professional groups were statistically significant (p=0.001), with medical professionals showing higher awareness (51.7%) compared to dental (35.7%), pharmacy (30.0%), nursing (26.7%) and allied health professionals (20.8%). When asked about the frequency of searching for research grants, 42.9% (n=171) of participants reported never searching for grants, 32.1% (n=129) searched occasionally (1-2 times per year), 17.9% (n=71) searched monthly and 7.1% (n=29) searched weekly. Participants were asked about the time taken to draft a research proposal. The results showed that 37.1% (n=149) took 1 month, 28.6% (n=114) took 2-3 months, 21.4% (n=86) took less than 1 month and 12.9% (n=51) took more than 3 months to draft a proposal. When asked about the important criteria for selection of research proposals by funding agencies, 45.7% (n=183) identified research methodology, 28.6% (n=114) identified innovation, 17.1% (n=68) identified relevance to national health priorities and 8.6% (n=35) identified budget justification as the most important criterion (Table1-4 - see PDF).

## Discussion:

This study aimed to assess the perception of healthcare researchers regarding funding opportunities of extramural research grants in India and identify challenges faced by health researchers in drafting grant proposals. The findings reveal a significant lack of awareness about funding agencies and opportunities among health professionals, particularly those outside the medical discipline. Only 48.6% of participants in our study were aware of extramural research funding agencies in India, which is concerning given the importance of research funding for advancing health sciences. This finding is consistent with previous studies that have reported limited awareness about research funding opportunities among health professionals in India [4, 7]. The higher awareness among medical professionals (68.3%) compared to dental (42.9%), pharmacy (35.0%), nursing (30.0%) and allied health professionals (25.0%) indicates a disparity in knowledge dissemination across health disciplines. This disparity could be attributed to several factors, including differences in research orientation in academic curricula, exposure to research activities and institutional emphasis on research. The medical curriculum in India traditionally places greater emphasis on research compared to other health disciplines, which may explain the higher awareness among medical professionals [4]. Among those aware of funding agencies, 82.9% could name only ICMR, indicating limited knowledge about the diverse funding landscape in India. The limited knowledge about funding agencies other than ICMR suggests a need for better dissemination of information about various funding opportunities available to health researchers in India. The finding that 85.7% of participants had never had opportunities to work on extramural grants is alarming and indicates a significant gap in research exposure and mentorship. This lack of opportunities was particularly pronounced among non-medical health professionals, with only 4.2-12.5% reporting such experiences compared to 25.0% of medical professionals. This disparity may contribute to the uneven development of research capacity across health disciplines in India. The limited opportunities to work on extramural grants could be attributed to several factors, including institutional research culture, availability of mentors with grant-writing experience and disciplinary focus on research. Previous studies have identified lack of mentorship and institutional support as major barriers to research engagement among health professionals in India [4, 7]. The findings of our study underscore the need for creating more opportunities for hands-on experience with extramural grants across all health disciplines. The finding that 74.3% of participants did not know the pattern of drafting research proposals highlights a significant skills gap among health professionals in India. This lack of knowledge about grant proposal writing was particularly evident among non-medical health professionals, with only 12.5-21.4% reporting familiarity with the process compared to 40.0% of medical professionals. This skills gap represents a major barrier to securing research funding and conducting independent research. The limited knowledge about grant proposal writing is consistent with previous studies that have identified lack of training in research methodology as a major barrier for health research in India [4]. Our findings extend this observation to other health disciplines and highlight the need for incorporating grant writing skills into the academic curriculum of all health professional programs. The limited awareness of career opportunities related to funding agencies (37.1% overall) indicates a narrow perspective on research career pathways among health professionals in India. This lack of awareness may limit the exploration of diverse career options in research and funding administration. The higher awareness among medical professionals (51.7%) compared to other disciplines suggests a difference in career guidance and exposure to research career pathways. The finding that 42.9% of participants never searched for research grants and only 7.1% searched weekly indicates limited proactive engagement with funding opportunities. This passive approach to seeking research funding may contribute to the underutilization of available resources and limit research productivity. The infrequent searching for grants could be attributed to lack of awareness, perceived complexity of grant application processes, or competing professional responsibilities. The finding that 37.1% of participants took 1 month to draft a research proposal, while 12.9% took more than 3 months, indicates variability in the efficiency of proposal development. The time taken to draft proposals may reflect familiarity with the process, availability of mentorship and institutional support for research activities. The longer duration reported by some participants may indicate challenges in understanding proposal requirements, developing research methodologies, or preparing budgets. The identification of research methodology (45.7%) as the most important criterion for selection of research proposals by funding agencies reflects an understanding of the technical aspects of grant evaluation. However, the limited emphasis on innovation (28.6%) and relevance to national health priorities (17.1%) suggests a potential gap in understanding the broader context of research funding decisions. Funding agencies typically consider multiple criteria, including scientific merit, innovation, relevance, feasibility and budget justification, when evaluating proposals [9, 10]. The findings of our study have significant implications for health research in India. The limited awareness of funding agencies and opportunities among health professionals, particularly those outside the medical discipline, suggests a need for targeted interventions to enhance knowledge and engagement. The disparities in awareness and experience across health disciplines highlight the need for more inclusive approaches to research capacity building. The lack of knowledge about grant proposal writing and limited experience with extramural grants indicate a need for incorporating research skills training into the academic curriculum of all health professional programs. Previous studies have emphasized the importance of research training for health professionals in India [4, 7] and our findings support the need for such training to include grant writing skills. The limited proactive engagement with funding opportunities suggests a need for creating a more supportive environment for research, including mentorship programs, institutional incentives for research engagement and streamlined application processes. Previous studies have identified institutional support as a key factor in promoting research engagement among health professionals [4, 7]. The challenges identified in our study are not unique to India but reflect broader issues in global health research. Studies from other low-and middle-income countries have reported similar challenges in research funding awareness and grant writing skills [8]. However, the disparities across health disciplines observed in our study may be more pronounced in India due to the historical emphasis on medical research and the relatively recent development of research capacity in other health disciplines. The global health research community has recognized the importance of building research capacity across all health disciplines to address complex health challenges [8]. In India, just a fraction of the little budget for dental health research goes into investigating ways to improve the public's oral health [11]. Our findings highlight the need for similar efforts in India to ensure that all health professionals can contribute to and benefit from research funding opportunities.

## Conclusion:

A critical gap in awareness and utilization of extramural research funding among Indian health professionals, especially in non-medical fields is shown. Integrating research grant writing into health curricula and expanding training initiatives are essential to strengthen research capacity. Enhancing awareness and inclusivity in funding access will enable India to better align its research efforts with national and global health priorities.

## Source of Funding:

NIL
